# Chromosome-Scale Assembly of the Complete Genome Sequence of Leishmania (Mundinia) enriettii, Isolate CUR178, Strain LV763

**DOI:** 10.1128/MRA.00575-21

**Published:** 2021-09-09

**Authors:** Hatim Almutairi, Michael D. Urbaniak, Michelle D. Bates, Vanete Thomaz-Soccol, Waleed S. Al-Salem, Rod J. Dillon, Paul A. Bates, Derek Gatherer

**Affiliations:** a Division of Biomedical & Life Sciences, Faculty of Health & Medicine, Lancaster University, Lancaster, United Kingdom; b Ministry of Health, Riyadh, Saudi Arabia; c Laboratório de Biologia Molecular, Programa de Pós Graduação em Engenharia de Bioprocessos e Biotecnologia, Universidade Federal do Paraná, Curitiba, Brazil; Vanderbilt University

## Abstract

Leishmania (Mundinia) enriettii is a parasitic kinetoplastid first isolated from a guinea pig in Brazil in 1946. We present the complete genome sequence of *L.* (*M.*) *enriettii*, isolate CUR178, strain LV763, sequenced using combined short-read and long-read technologies. This will facilitate a greater understanding of the genome diversity within *L.* (*M.*) *enriettii*.

## ANNOUNCEMENT

Leishmania enriettii was first isolated from a guinea pig (Cavia porcellus) in Brazil in 1946 and formally described 2 years later (1). The group of related species previously known as the *L. enriettii* complex is now referred to as the subgenus *Mundinia* (2, 3). A previous genome sequence of *L.* (*M.*) *enriettii*, isolate LEM3045 (MCAV/BR/1995/CUR3), was assembled but with a large number of unplaced contigs, higher gap content, and relatively lower *N*_50_ value compared to other *Leishmania* genomes (2). Additional *L.* (*M.*) *enriettii* isolates have been associated with leishmaniasis lesions in guinea pigs in the Curitiba Metropolitan Region of southern Brazil, 50 years after the first case (4, 5). We report here the complete genome assembly and annotation of *L.* (*M.*) *enriettii*, isolate CUR178, strain LV763 (WHO code MCAV/BR/2001/CUR178;LV763). This will contribute to our understanding of the biology of *L.* (*M.*) *enriettii* and the genomic diversity existing in this species.

We obtained intracellular amastigote parasites from six naturally infected guinea pigs from the rural area of Mandirituba (state of Paraná, Brazil). The parasites were grown using an *in vitro* culture system previously developed for Leishmania (Mundinia) orientalis axenic amastigotes (6) in Schneider’s insect medium at 26°C as promastigotes, then in M199 medium supplemented with 10% fetal calf serum (FCS), 2% stable human urine, 1% basal medium Eagle vitamins, and 25 μg/ml gentamicin sulfate, with subpassage to fresh medium every 4 days to sustain the parasite growth and viability. DNA was extracted and purified using a Qiagen DNeasy blood and tissue kit using the spin column protocol, according to the manufacturer’s instructions. The extracted DNA concentration was assessed using a Qubit fluorometer, microplate reader, and agarose gel electrophoresis. All sequencing libraries were based on the same extracted DNA sample to avoid any inconsistency.

Short-read library construction and sequencing were contracted to (i) BGI (Shenzhen, China) for DNBSEQ libraries, producing paired-end reads (270 bp and 500 bp) using the Illumina HiSeq platform, and (ii) Aberystwyth University (Aberystwyth, UK) for TruSeq Nano DNA libraries, producing paired-end reads (300 bp) using the Illumina MiSeq platform. We performed long-read library preparation and sequencing according to the Nanopore protocol (SQK-LSK109) on R9 flow cells (FLO-MIN106). The read quality was assessed using MultiQC (7).

We assembled the long reads using Flye (8), with default parameters, to generate chromosome-scale scaffolds. Then, using Minimap2 (9) and SAMtools (10), we mapped the short reads onto the assembled scaffolds to correct erroneous bases within long reads and create consensus sequences. After polishing the assembly with Pilon (11), another round of consensus short read mapping was performed. Then, we removed the duplicated contigs and sorted the remainder according to length using Funannotate (12, 13). Finally, we separated the chimeric sequences and performed scaffolding using RaGOO (14) with the Leishmania major strain Friedlin genome (GenBank accession number GCA_000002725.2) (15) as a reference guide, aligning all 36 chromosomes for our assembly, thereby also determining the chromosome ends to be complete, with the exception of 18 unplaced contigs totaling 76,607 bp.

**FIG 1:**
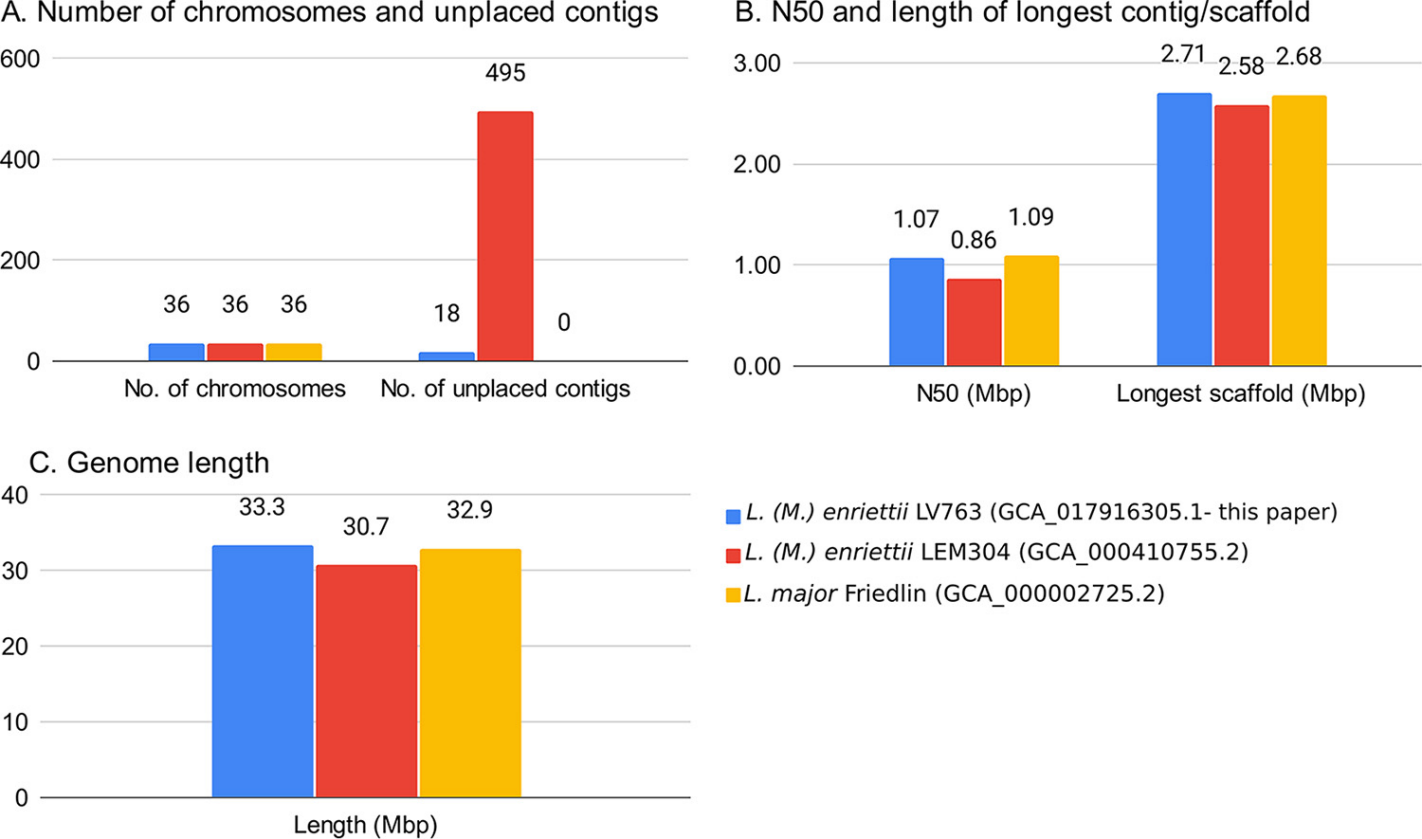
Assembly comparison of *L.* (*M.*) *enriettii* LV763 with *L.* (*M.*) *enriettii* LEM3045 and L. major Friedlin.

The analysis workflow for assembly, repeat masking, and annotation was performed using Snakemake (16); it is available online for reproducibility purposes (https://github.com/hatimalmutairi/LGAAP), including the software versions and parameters used (17). Figure 1 compares our assembly with other complete genomes.

We assessed the assembly completeness using BUSCO (18), with the lineage data set for the phylum *Euglenozoa*, containing 130 single-copy orthologs from 31 species, and we found that 123 of these orthologs were present (94.6% completeness). We carried out functional annotation and prediction using the MAKER2 (19) annotation pipeline in combination with AUGUSTUS (20) gene prediction software, with the predictor trained on Leishmania tarentolae. Table 1 shows additional summary metrics for the sequencing, assembly, and annotation.

**TABLE 1:** Detailed summary metrics of the genome sequencing, assembly, and annotation for *L*. (*M.*) *enriettii* LV763

Feature(s)	Metric(s)
Total no. of reads	26,789,424
No. of MiSeq reads	5,060,124
No. of HiSeq reads	20,936,270
No. of MinION reads (read *N*_50_ [bp])	793,030 (12,070)
No. of bases (Gb)	19.41
Genome coverage (×)	271.8
Total no. of scaffolds	54
Genome size (bp)	33,318,864
*N*_50_ (bp)	1,075,649
GC content (%)	59.60
No. of Ns (% of genome)	380 (0.001)
No. of genes	8,353
Gene density (no. of genes/Mb)	250.7
No. of exons	8,584
Mean gene length (bp)	1,897
Total length of CDSs^*a*^ (Mb) (% of genome)	15.46 (46.40)

a CDSs, coding DNA sequences.

### Data availability.

The assembly and annotations are available under GenBank assembly accession number GCA_017916305.1. The master record for the whole-genome sequencing project is available under accession number JAFHKP000000000.1. The raw sequence reads are available under accession number PRJNA691534.
